# Uncovering the Role of Indian Medicinal Botanicals in COVID-19 Prevention and Management: A Review

**DOI:** 10.7759/cureus.75920

**Published:** 2024-12-18

**Authors:** D. Meena S Rao, Jyotsana Mishra, Sarwade Vasudeo Damodar, Jalindarnath Gajendra Bagal, Vinayaka K S, Renju Ammu Joseph, Theresa Karra, Ruchita Shrivastava

**Affiliations:** 1 Department of Botany, R. K. Talreja College of Arts, Science and Commerce, Thane, IND; 2 College of Forestry, Mahatma Gandhi University of Horticulture and Forestry, Sankra-Patan, IND; 3 Department of Chemistry, Jamkhed Mahavidyalaya Jamkhed, Jamkhed, IND; 4 Department of Botany, Eknath Sitaram Divekar College, Varvand, IND; 5 Department of Botany, Sri Venkataramana Swamy College, Vidyagiri, IND; 6 Department of Botany, Fatima Mata National College, Kollam, IND; 7 Department of Zoology, St. Joseph's University, Bengaluru, IND; 8 Department of Botany, Govt. Home Science PG College, Narmadapuram, IND

**Keywords:** anti-inflammatory, antioxidant, ayurveda, covid-19, immune-boosting, medicinal plants

## Abstract

Indian traditional medicine, based on Ayurveda and Siddha, has become one of the global searches for complementary approaches to conventional interventions during the COVID-19 pandemic. This review presents the antiviral, immune-boosting, and anti-inflammatory properties of some medicinal key plants such as Tulsi (*Ocimum sanctum*), Neem (*Azadirachta indica*), Ashwagandha (*Withania somnifera*), Amla (*Emblica officinalis*), and Giloy (*Tinospora cordifolia*). Tulsi appears to inhibit viral replication, Neem increases immune cell synthesis, while Ashwagandha regulates inflammation and stress responses. Vitamin C-rich Amla increases immune defense while also providing protection against oxidative stress and Giloy modulates immune response and its activity, acting as an overall resilience against infection. However, the clinical integration of these plants into mainstream healthcare is hindered by the absence of robust clinical trials, standardization of phytochemicals, and the absence of global standard protocols. In order to establish safety and efficacy, substantial research is needed, including large-scale randomized clinical trials and sophisticated bioinformatics techniques. Indian medicinal plants provide innovative, sustainable, and holistic solutions to global health crises, such as the COVID-19 pandemic, by bridging traditional knowledge with modern scientific frameworks.

## Introduction and background

The COVID-19 pandemic unveiled shortcomings of current healthcare systems and led to a worldwide search for alternative or complementary therapeutic solutions. Traditional Indian health systems, in particular those based on Ayurveda and Siddha, have received special attention because they emphasize whole-body health, immune modulation, and reliance on natural remedies. Such systems are based deeply on ancient texts such as Charaka Samhita and Sushruta Samhita which have been formulated in detail for treating various ailments, such as respiratory and infectious diseases [1]. The antiviral properties of Tulsi (*Ocimum sanctum*) as per Ayurveda have been previously validated recently through its efficacy in suppressing viral replication by bioactive compounds eugenol and ursolic acid [2].

Ayurveda and Siddha are two different ancient Indian systems of medicine, apart from the fact that they are different in their origin, philosophy, and practice. Dating back to the Vedic period, Ayurveda embraces that its three doshas (Vata, Pitta, and Kapha) must be balanced with herbal medicine, yoga, and dietary practices as well [3]. On the other hand, Siddha medicine, the traditional health system that is mainly practiced in south India, draws its base upon the Tamil culture and has the understanding regarding the five-elemental theory in which health is related to the harmony of five principal elements (earth, water, fire, air, and space) [4]. Moreover, Siddha makes use of mineral-based formulations along with plant-based remedies. In addition to these, other traditional systems such as Unani that were introduced to India through Persian influences focus on the balance of bodily humors and folk medicine based on locally available herbal practices and oral traditions are also part of the big and diverse medicinal heritage of India [5]. These systems, working together, form a holistic view of how to better respond to health challenges, including the COVID-19 pandemic.

Contemporary research methodologies are largely influenced by traditional knowledge. For example, Ashwagandha (*Withania somnifera*) mentioned in ancient Ayurvedic text was used to modulate the immune system and its effects on stress reduction. However, modern studies have been inspired by these effects and have isolated withanolides, the active compounds involved, and have also studied them as a means to manage viral infections, including COVID-19 [6]. Just as Neem (*Azadirachta indica*) has been the subject of investigations due to the antiviral and immune-enhancing properties of its bioactive compounds (nimbidin, nimbin) [7], so are bioactive compounds from other medicinal plants of interest. These examples give an idea about the vital role of traditional knowledge in modern pharmacological development.

The pandemic highlighted the need to strengthen immunity and control inflammation, symptoms that traditional medicine focuses on as therapeutic. A trending spree of demand for herbal formulations like Chyawanprash (rich in Amla and Ashwagandha) and the use of Tulsi-rich teas and decoctions indicates a societal shift toward preventive healthcare. Furthermore, the scientific evaluation of these remedies was accelerated with computational modeling to assess the efficacy of phytochemicals and clinical trials to validate safety. Reinforcement of these efforts suggests youthfulness for integrative medicine as a tool for dealing with global health crises while at the same time keeping traditional wisdom intact.

## Review

Current understanding: established theories and recent advances

The outbreak of COVID-19 demonstrated the weaknesses of traditional medical systems to control an unprecedented world health crisis. The interest in alternative and complementary therapies, including traditional medicine, arose due to this situation. Traditional medicine has been recognized by the World Health Organization (WHO) as an effective means to manage symptoms, and research into its use should be conducted [8]. Because of bioactive compounds with antiviral, immune-boosting, anti-inflammatory, and antioxidant properties, Indian medicinal plants have emerged as strong candidates.

Tulsi (Ocimum sanctum) and Its Antiviral Properties

In traditional Indian medicine, tulsi or holy basil has long been revered for its wide-ranging therapeutic properties. In preclinical studies, its phytochemicals, eugenol, ursolic acid, and oleanolic acid, have been found to exhibit antiviral activity. For instance, Bhattacharya et al. (2024) demonstrated that Tulsi extracts inhibited viral replication and modified immune response, particularly with respiratory infections [2]. A recent study by Jamshidi and Cohen (2017) examined Tulsi’s potential to fight viral infections, including respiratory viruses [9]. Clinical evidence of its efficacy against SARS-CoV-2 is lacking, and larger randomized controlled trials (RCTs) are needed to determine its place in the management of COVID-19.

Neem (Azadirachta indica) and Its Immune-Boosting Effects

Indian traditional medicine has its cornerstone in neem, often referred to as 'the village pharmacy'. Nimbin and nimbidin are well-known bioactive compounds that stimulate the synthesis of immune cells and cytokines, enhancing the body’s defense mechanisms. The study by Wylie and Merrell (2022) found that neem has antiviral and immune-boosting properties, and it has the potential to lower the viral load in a laboratory setting [10]. Nevertheless, further work is required to extrapolate these results to clinical applications, as human trials are needed to validate these findings. Moreover, neem’s hepatotoxic potential in some settings demands clinical cautiousness.

Ashwagandha (Withania somnifera) and Its Adaptogenic Qualities

Ashwagandha is recognized for its 'adaptogenic' properties, helping the body deal with stress and clean up physiological equilibrium. A study done by Wankhede et al. (2015) shows that the withanolides present in Ashwagandha have immunomodulatory effects and improved immune function and reduced inflammation in preclinical settings [11]. In silico studies have recently suggested that Ashwagandha compounds may interact with SARS CoV 2 viral proteins and may inhibit viral entry [12]. However, these findings are mostly theoretical and need to be validated in the clinic to see if they can be used to treat COVID-19. Moreover, the stress-reducing properties of Ashwagandha could be beneficial to general health; however, its use during acute viral infection is yet to be explored in RCTs.

Amla (Emblica officinalis) and Its Rich Vitamin C Content

Amla (Indian gooseberry) is a rich source of Vitamin C, which is absolutely necessary for providing nutrition to the immune system and enhancing immunity power. Amla extracts have also been proven in clinical studies to reduce oxidative stress and inflammation and support overall immune health [13]. During the COVID-19 pandemic, Amla played a notable role in immunity and is commonly found in formulations like Chyawanprash. While Amla supplementation shows great promise as a potentially useful immune modulator to assist COVID-19 patients, there is still limited direct evidence of its influence on outcomes. A study by Varnasseri et al. (2022) explored the potential of Amla in managing viral infections; however, more clinical evidence is needed to confirm its efficacy, particularly in COVID-19 cases [14].

Giloy (Tinospora cordifolia) as an Immunomodulator

In Ayurveda, Giloy is very much valued for its immunomodulatory effects. A study by Devi (2021) has shown that active compounds, such as beta glucans, increase immune cell activity [15]. Small-scale studies during the COVID-19 pandemic have shown that Giloy supplementation improves immune resilience in mild cases of the disease. Nevertheless, these studies were often lacking control groups and were not based on standardized protocols. To sustain Giloy’s role in managing viral infections, high-quality RCTs are necessary. In a recent trial, published by Balkrishna et al. (2021), Giloy showed promise as an immune support complement, although the results must be validated in more clinical trials [16].

Moringa (Moringa oleifera) and Its Nutrient-Packed Leaves

The immunomodulatory properties of Moringa are due to its rich vitamin, mineral, and antioxidant content. The bioactive compounds quercetin and chlorogenic acid have been shown to increase immune responses to viral infections [17]. Giugliano et al. (2024) recently observed that Moringa extracts diminished inflammation and oxidative stress markers in preclinical models of respiratory infections. Moreover, Moringa has been tested in vitro against some viruses [18]. Direct evidence of the efficacy of its application in COVID-19 is however lacking, and further studies are needed to translate the above findings into clinical matters.

Critical analysis: gaps, controversies, and limitations

Insufficient Clinical Evidence

Large-scale RCTs for medicinal plant studies are still limited, despite promising laboratory studies. For example, Tulsi possesses antiviral ability against respiratory pathogens but does not have clinical validation with SARS-CoV-2 [2]. With limited funding sources, the absence of standardized protocols, and difficulties in recruiting diverse patient groups, conducting these trials presents significant challenges. Bridging the gaps between government agencies, traditional medicine institutions, and pharmaceutical companies could help address these issues. This lack of such trials impedes the acceptance of these plants in evidence-based medicine. The investigation into efficacy and safety under standardized conditions should be carried out with high-quality trials in the future.

Standardization Challenges

Medicinal plant bioactive compound consistency is highly dependent on cultivation, harvesting, and preparation methods, and the therapeutic effectiveness of medicinal plants is hindered as a result. For example, the vitamin C content of Amla depends on geographic and environmental factors [19]. Overcoming these inconsistencies requires that globally accepted quality control and phytochemical fingerprinting standards be developed.

In recent years, with the aid of state-of-the-art technologies including bioinformatics and machine learning, recent advancements in plant-based therapeutics have been made to identify and optimize bioactive compounds. For example, AI models are now being deployed to predict the potency of bioactive compounds from plant extracts from large datasets of phytochemical properties. Molecular docking studies as computational biology tools aid in discovering the antiviral potential of compounds like ursolic acid in Tulsi or withanolides in Ashwagandha [20]. AI algorithms are combined with phytochemical databases, such as the Indian Medicinal Plants Database (IMPD) to improve data retrieval and compound prediction enabling a more precise, albeit less time-consuming, drug discovery. Such innovations bridge the traditional knowledge and modern pharmacological research and enhance the modern context for plant-based remedies around contemporary medicine.

Integration of Traditional and Modern Medical Approaches

Holistic and personalized care were hallmarks of traditional approaches, whereas modern medicine has adopted a more reductionist methodology. Bridging this gap requires interdisciplinary frameworks that integrate traditional plant-based therapies into contemporary treatment protocols while adhering to strict validation processes [21]. However, intellectual property rights continue to raise ethical concerns, particularly regarding the commodification of indigenous knowledge and the risk of traditional knowledge holders receiving unequal benefits. They also have to address the practical challenge of regulatory approval and quality control. The ethical integration of traditional remedies into modern medicine requires transparency and equitable collaboration with indigenous communities to prevent exploitation.

Challenges and Risks in Medicinal Plant Use

Medicinal plants are used without professional advice widely and can lead to adverse effects. Excessive consumption of neem or Giloy is known to have caused hepatotoxicity in some cases. Another problem is adulteration of herbal products, containing unsafe levels of excess heavy metals or contaminants. We need strengthened regulatory frameworks and better education on the use of this technology safely. Safe usage can’t be promoted without public awareness and education campaigns.

Innovative Perspectives in Plant-Based Therapeutics

Plant compounds are being explored for synergies with conventional therapies during the COVID-19 pandemic. Combining bioactive compounds of neem or Ashwagandha with antiviral drugs is shown to enhance the treatment efficacy and decrease side effects [22]. Beyond that, artificial intelligence (AI) and machine learning (ML) can be used to analyze plant-based pharmacological data, accelerate drug discovery, and optimize dosage formulations. For instance, Ashwagandha compound interactions with SARS-CoV-2 proteins are being predicted through AI-driven simulations. The screening of large libraries of medicinal plants for potential antiviral activity by neural networks is similarly done to reduce the time and cost of drug discovery. Supply chain management can also be integrated with AI to ensure traceability and quality control of medicinal plant products. Such computational tools can also be integrated to uncover novel therapeutic pathways and predict synergistic effects between plant compounds and synthetic drugs.

Applications and implications: practical uses and technological advancements

During the COVID-19 pandemic, Indian medicinal plants have been used in several practical applications to strengthen immunity, maintain respiratory health, and manage associated symptoms. Traditional use of these plants has led to their introduction into formulations, remedies, and preventive strategies.

Indian medicinal plants have therapeutic potential in the management of COVID 19 as highlighted in Table 1. The plants have varied properties including antiviral, immune boosting, anti-inflammatory, and antioxidant. Vasaka and licorice are useful for respiratory health and reducing congestion and inflammation, and Tulsi, neem, and Giloy support immunity and may inhibit viral replication. Ashwagandha and holy basil are adaptogenic plants that help reduce stress and maintain overall well-being. This provides indicia of the importance of these plants as part of holistic health strategies for viral infections including COVID-19.

**Table 1:** Medicinal Plants With Potential Roles Against COVID-19

Medicinal Plant	Key Properties	Role Against COVID-19	Reference
Tulsi (*Ocimum sanctum*)	Antiviral, Immunomodulatory	Inhibits viral replication, supports immune response	[2]
Neem (*Azadirachta indica*)	Immune-boosting, Antiviral	Enhances immunity, potential antiviral activity	[7].
Ashwagandha (*Withania somnifera*)	Adaptogenic, Immunomodulatory	Supports immune function, helps manage stress	[11]
Amla (*Emblica officinalis*)	Rich in Vitamin C, Antioxidant	Boosts immune response, reduces oxidative stress	[19]
Giloy (*Tinospora cordifolia*)	Immunomodulatory	Modulates the immune system, aids defense against infections	[15]
Moringa (*Moringa oleifera*)	Nutrient-rich, Antioxidant	Enhances overall health, combatting oxidative stress	[17]
Vasaka (*Adhatoda vasica*)	Expectorant, Bronchodilatory	Aids in clearing mucus, supports respiratory function	[23]
Licorice (*Glycyrrhiza glabra*)	Anti-inflammatory, Soothing	Reduces respiratory discomfort, supports lung health	[24]
Pushkarmool (*Inula racemosa*)	Bronchodilatory, Respiratory support	Relaxes airways, eases respiratory congestion	[25]
Turmeric (*Curcuma longa*)	Antioxidant, Anti-inflammatory	Combats oxidative stress, reduces inflammation	[26]
Ginger (*Zingiber officinale*)	Anti-inflammatory, Immune support	Soothes respiratory distress, enhances immune function	[27]
Holy Basil (*Ocimum sanctum*)	Adaptogenic, Antioxidant	Reduces stress, supports overall well-being	[28]

Ayurvedic formulations

Ayurvedic products formulated using medicinal plants have gained widespread use for immunity enhancement and general health support (Table 2).

**Table 2:** Ayurvedic Formulations for COVID-19 Management

Formulation	Key Ingredients	Main Active Components	Chemical Formula	Role in COVID-19	Reference
Chyawanprash	Amla (Emblica officinalis), Ashwagandha (Withania somnifera), Giloy (Tinospora cordifolia), Pippali (Piper longum)	Vitamin C, withanolides, beta-glucans, piperine	C6H8O6 (Vitamin C), C28H38O6 (Withaferin A), C18H32O16 (Beta-glucans), C17H19NO3 (Piperine)	Enhances immunity, reduces oxidative stress, and supports respiratory health. Provides immunomodulatory effects and improves bioavailability of herbs.	[29]
Giloy Ghanvati	Giloy (Tinospora cordifolia)	Beta-glucans, alkaloids (berberine, tinosporine)	C18H32O16 (Beta-glucans), C20H18NO4 (Berberine)	It increases the immune response by activating macrophages and reducing inflammation. Good for mild symptoms such as fever and fatigue.	[16]
Ayush Kwath	Tulsi (Ocimum sanctum), Dalchini (Cinnamomum verum), Sunthi (Zingiber officinale), Kali Mirch (Piper nigrum)	Eugenol, cinnamaldehyde, gingerol, piperine	C10H12O2 (Eugenol), C9H8O (Cinnamaldehyde), C17H26O4 (Gingerol), C17H19NO3 (Piperine)	Antiinflammatory, antiviral, and antioxidant properties. It supports immunity, prevents respiratory symptoms, and helps herb absorption.	[30]
Sitopaladi Churna	Vanshlochan (Bambusa arundinacea), Pippali (Piper longum), Ela (Elettaria cardamomum), Dalchini (Cinnamomum verum)	Silica, piperine, cinnamaldehyde, cineole	SiO2 (Silica), C17H19NO3 (Piperine), C9H8O (Cinnamaldehyde), C10H18O (Cineole)	Helps with cough, throat irritation and congestion. It relieves mild respiratory symptoms.	[31]
Trikatu Churna	Sunthi (Zingiber officinale), Pippali (Piper longum), Maricha (Piper nigrum)	Gingerol, piperine, chavicine	C17H26O4 (Gingerol), C17H19NO3 (Piperine), C17H19NO3 (Chavicine)	It improves digestion, strengthens immunity and acts as an enhancer of bioavailability of other formulations.	[32]
Dashmoolarishta	Ten medicinal roots, including Bilva (Aegle marmelos), Gambhari (Gmelina arborea), Shalaparni (Desmodium gangeticum)	Alkaloids, flavonoids, tannins	Variable (Alkaloids), C15H10O2 (Flavonoids), Variable (Tannins)	Helps fortify the respiratory system, decreases inflammation and relieves weakness and fatigue, especially during post COVID recovery.	[33]
Anu Taila	Jivanti (Leptadenia reticulata), Yashtimadhu (Glycyrrhiza glabra), Bala (Sida cordifolia)	Glycyrrhizin, phytosterols, alkaloids	C42H62O16 (Glycyrrhizin), Variable (Phytosterols), Variable (Alkaloids)	It maintains nasal hygiene, prevents respiratory infections and reduces nasal inflammation.	[34]
Panch Tulsi Drops	Extracts of five varieties of Tulsi (Ocimum sanctum, Ocimum gratissimum, Ocimum canum, Ocimum basilicum, Ocimum citriodorum)	Eugenol, ursolic acid, rosmarinic acid	C10H12O2 (Eugenol), C30H48O3 (Ursolic Acid), C18H16O8 (Rosmarinic Acid)	It inhibits viral replication and inflammation, boosts immunity, reduces oxidative stress and supports respiratory health.	[35]

Respiratory support

Respiratory health has been a critical focus during COVID-19, and Indian medicinal plants have been employed effectively in managing respiratory ailments.

*Adhatoda vasica *(Vasaka) is a widely used herb in the management of bronchitis, asthma, and chronic cough. The active compounds, vasicine and vasicinone, are natural expectorants and bronchodilation, helping to clear out the respiratory tract and make breathing easier. In addition, these compounds prevent airway inflammation, making Vasaka very effective in combating congestion and wheezing during viral respiratory infections such as COVID-19 [23].

Mulethi (*Glycyrrhiza glabra*) or licorice root is famous for its soothing properties on irritated mucous membranes of the respiratory tract. Glycyrrhizin, an active compound, has an antiviral effect that inhibits viral replication and reduces inflammation in the lungs. Mulethi has a special place in treating sore throats, persistent coughs, and respiratory irritation caused by COVID-19 [24].

Pushkarmool (*Inula racemosa*) is a potent herb for promoting respiratory health. It is an herb that is quite bronchodilatory and anti-inflammatory. Its active compounds, alantolactone, and isoalantolactone, relax the smooth muscles of the airways, helping increased airflow and increasing lung capacity. Pushkarmool makes these actions effective in treating respiratory congestion and reducing inflammation in the bronchial passages, as in asthma and COPD, aggravated by viral infections [25].

Pippali (*Piper longum*), a long pepper, is used as a mucolytic (mucous-breaking) herbal treatment for the removal of thick mucus from the respiratory system. Its active compound, piperine is a bronchodilator that works by relaxing airway muscles, increasing airflow, and relieving nasal and chest congestion. Pippali enhances the bioavailability of other respiratory-supportive herbs, thereby amplifying the overall efficacy of herbal formulations [36].

Herbal teas and decoctions

Herbal teas and decoctions incorporating Indian medicinal plants have been widely consumed for their therapeutic effects on respiratory and immune health.

Tulsi Tea

It is a widely used herbal remedy for health of the respiratory system, which includes Tulsi (*Ocimum sanctum*), ginger (*Zingiber officinale*), and honey. Tulsi tea does that by stopping viral replication and calming inflamed airways. Ginger makes it more therapeutic by reducing swelling in the respiratory tract and enhancing immune responses. Like a natural demulcent, honey coats and soothes the throat providing relief of irritation and mild cough. Tulsi tea is an effective and soothing remedy for sore throat, congestion, and mild respiratory infections [28].

Lung Support Decoctions

Mulethi (*Glycyrrhiza glabra*), thyme (*Thymus vulgaris*), and ginger (*Zingiber officinale*) are used in lung support decoctions to help relieve chronic cough, congestion, and respiratory discomfort. Mulethi calms down irritated mucous membranes and relieves irritation and inflammation in the throat. Thyme is a natural expectorant, helping to clear mucus from the airways and ginger helps to reduce inflammation in the respiratory tract. Together these ingredients can improve lung function and ease symptoms of respiratory distress [24].

Trikatu Tea

The Ayurvedic formulation Trikatu Tea is made from Sunthi (*Zingiber officinale*), Maricha (*Piper nigrum*), and Pippali (*Piper longum*). This tea is great for clearing the mucus buildup and nasal congestion and increasing airflow. As Trikatu tea is an active one, it contains the decongestant and bronchodilator agent, more commonly known as gingerol and piperine [32,36].

Vasaka Decoction

Vasaka (*Adhatoda vasica*) is another such herbal remedy for respiratory health. The decoction loosens mucus, clears the airways, and reduces inflammation in the lungs. It is especially useful in relieving chest congestion, wheezing, and shortness of breath due to respiratory infections. Vasaka is an expectorant and anti-inflammatory herb that is important for treating asthma-like symptoms as well as relieving congestion during viral infections [23].

Home remedies

Simple home remedies using Indian medicinal plants have also been widely adopted to manage mild symptoms and support preventive care.

Steam Inhalation With Eucalyptus

Steam inhalation with Eucalyptus oil is a popular remedy to relieve nasal and chest congestion. Due to its antimicrobial properties, eucalyptus oil helps in reducing inflammation of the respiratory tract and clearing airways [37].

Honey and Ginger Syrup

Honey and ginger blend in between relieving throat irritation and cough. Honey has a natural demulcent action on the throat, and ginger bioactive compounds such as gingerol have anti-inflammatory and antioxidant effects [27].

Neem Water Gargle

A traditional way to reduce the microbial load in the throat is gargling with warm water containing neem (*Azadirachta indica*) leaves. Neem has antiviral and antibacterial properties which promote oral health and prevent infections in the upper respiratory tract [7].

Future exploration and advancement

Indian medicinal plants hold immense promise in addressing global health challenges, but their full potential can only be realized through a strategic, evidence-based approach. The following steps highlight the key areas for further investigation and development.

Clinical Research

Rigorous clinical research by which the therapeutic efficacy of Indian medicinal plants needs to be established is an essential part. To determine their safety and their effectiveness against viral infections, including COVID-19, large-scale, randomized controlled trials (RCTs) are needed. For instance, while a large body of preclinical work exists demonstrating the antiviral potential of plants like Tulsi (*Ocimum sanctum*) and neem, (*Azadirachta indica*), strong clinical data are lacking [9,10]. These findings can be validated by RCTs and accepted by evidence-based medicine. On the other hand, small trials have shown promise for the use of Giloy (*Tinospora cordifolia*) as a booster of immunity, but these studies need confirmation of its safety and efficacy in larger studies [38].

Standardization and Quality Control

Reproducibility and consistency of therapeutic effects are dependent on standardization. Plant cultivation, harvesting, and preparation methods are highly variable such that outcomes tend to be inconsistent. Clearly defined formulations with defined dosages, quality control procedures, and demonstrated preparation protocols are essential to the development of standardized ones. Phytochemical fingerprinting and bioassay-guided fractionation can help identify and quantify active compounds to ensure uniformity across batches [39]. Moreover, it will enhance supply chain transparency as well as quality checks to build customer loyalty as well as regulatory approval.

Integrative Medicine Models

The integration of modern medicine with traditional medical remedies can be a holistic approach to healthcare. Collaboration frameworks for establishing collaboration bridges between traditional and modern medicine practitioners are necessary. It can breed new, integrated treatment protocols that take advantage of the strengths of each system. For instance, combining Ashwagandha (*Withania somnifera*) and conventional stress management therapies should demonstrate advantages regarding immune resilience and overall well-being [11]. Moreover, combining neem-based formulations with antiviral drugs could enhance the therapeutic effect synergistically.

Mechanistic Studies

The molecular and pharmacological mechanisms of bioactive compounds in medicinal plants must be understood for their advancement of therapeutic applications. Specific pathways through which these compounds exert their effects, such as antiviral, anti-inflammatory, and immunomodulatory, should be identified for research. Studies of turmeric (*Curcuma longa*) curcumin have found its ability to regulate cytokine storms and reduce oxidative stress in severe COVID-19 cases [26]. Like Giloy, the immunomodulatory effects of Giloy are also credited to its ability to boost the activity of macrophages and natural killer cells, but more investigation is needed to determine its mechanisms [15].

Public Awareness and Education

Public health campaigns and well-advised guidance from experts should be given to the safe and informed use of medicinal plants. Self-medication is common among many, and these plants do not have any adverse effects or do not interact with conventional drugs. However, it is critical to educate the public about proper dosing as well as potential side effects and to seek healthcare provider input [26]. Government and non-government organizations should also work together to disseminate correct information on the therapeutic benefits and limitations of Indian medicinal plants [40].

With brilliant promise in alleviating global health challenges, Indian medicinal plants need a strategic, evidence-based approach to gain their full potential. Establishing global guidelines for the integration of these remedies into healthcare systems requires collaboration with international organizations like the WHO. Transparency in research needs to improve and to facilitate collaboration among researchers and policymakers, a unified, open-access database for clinical trial data and bioactive compound profiles should be created. Funding large-scale clinical trials, incentivizing innovation, and regulatory frameworks on medicinal plants that ensure the safe and effective use of medicinal plants must be seen as policy interventions [40]. AI and ML can also make further revolutions in research by predicting compound efficacy, discovering synergistic combinations with synthetic drugs, and assuring standard formulations [20]. A phased roadmap, starting with regional validation and ending with global regulatory approval will pave the way for mainstream adoption of these remedies.

## Conclusions

Traditional systems such as Ayurveda and Siddha have shown remarkable potential of Indian medicinal plants in addressing global health challenges, like COVID-19. During the pandemic, the antiviral, immune-boosting, and anti-inflammatory properties of the bioactive compounds of Indian medicinal plants proved invaluable. Preclinical results from plants like Tulsi (*Ocimum sanctum*), Neem (*Azadirachta indica*), Licorice (*Glycyrrhiza glabra*), Ginger (*Zingiber officinale*), Pushkarmool (*Inula racemosa*), Ashwagandha (*Withania somnifera*), and Giloy (*Tinospora cordifolia*) have shown inhibiting viral replication, enhancing the activity of immune cells, and reducing inflammation. Nevertheless, difficulties with the lack of robust clinical trials and standardized protocols prevent the full integration of these biomarkers into evidence-based medicine. The article highlights the essential need for rigorous scientific validation, i.e., large-scale randomized clinical trials, phytochemical standardization, and quality control to the fulfillment of effective and safe utilization of these plants. Furthermore, AI and bioinformatics integration are proposed to accelerate drug discovery and formulate drugs for therapeutic use. For global adoption, these plants must be promoted to be used safely and in an informed way through educational efforts, as well as ethical frameworks to bridge traditional and modern medicine. The combination of ancient knowledge and modern science can offer new, sustainable, and holistic solutions to future health emergencies through Indian medicinal plants and thus initiate integrative healthcare systems all over the world.
